# Tuberous Sclerosis Complex Surveillance and Management: Recommendations of the 2012 International Tuberous Sclerosis Complex Consensus Conference

**DOI:** 10.1016/j.pediatrneurol.2013.08.002

**Published:** 2013-10

**Authors:** Darcy A. Krueger, Hope Northrup

**Affiliations:** a Division of Neurology, Department of Pediatrics, Cincinnati Children’s Hospital Medical Center, University of Cincinnati College of Medicine, Cincinnati, Ohio; b Division of Medical Genetics, Department of Pediatrics, University of Texas Medical School at Houston, Houston, Texas

**Keywords:** tuberous sclerosis, surveillance, treatment, management, guideline

## Abstract

**BACKGROUND:**

Tuberous sclerosis complex is a genetic disorder affecting every organ system, but disease manifestations vary significantly among affected individuals. The diverse and varied presentations and progression can be life-threatening with significant impact on cost and quality of life. Current surveillance and management practices are highly variable among region and country, reflective of the fact that last consensus recommendations occurred in 1998 and an updated, comprehensive standard is lacking that incorporates the latest scientific evidence and current best clinical practices.

**METHODS:**

The 2012 International Tuberous Sclerosis Complex Consensus Group, comprising 79 specialists from 14 countries, was organized into 12 separate subcommittees, each led by a clinician with advanced expertise in tuberous sclerosis complex and the relevant medical subspecialty. Each subcommittee focused on a specific disease area with important clinical management implications and was charged with formulating key clinical questions to address within its focus area, reviewing relevant literature, evaluating the strength of data, and providing a recommendation accordingly.

**RESULTS:**

The updated consensus recommendations for clinical surveillance and management in tuberous sclerosis complex are summarized here. The recommendations are relevant to the entire lifespan of the patient, from infancy to adulthood, including both individuals where the diagnosis is newly made as well as individuals where the diagnosis already is established.

**CONCLUSIONS:**

The 2012 International Tuberous Sclerosis Complex Consensus Recommendations provide an evidence-based, standardized approach for optimal clinical care provided for individuals with tuberous sclerosis complex.

## Introduction

The clinical manifestations of tuberous sclerosis complex (TSC) are highly diverse in both organ system involvement and severity. Any organ system can be involved, with some more prevalent during infancy and childhood and others more likely to affect individuals as adults.^1^ Birth incidence is estimated to be 1:5800.^2^ Many manifestations can be life-threatening and appropriate surveillance and management is necessary to limit morbidity and mortality in this disease. Appropriate management is also crucial for optimal quality of life of affected individuals and requires coordination of care among medical specialties and from childhood to adulthood on a regular basis and especially during the critical transition from pediatric to adult health care services.

In 1998, the National Institutes of Health sponsored the first Tuberous Sclerosis Complex Consensus Conference to develop recommendations for diagnosis and clinical management of patients affected by TSC.^3,4^ At that time, the two known genes responsible for TSC cases had been identified but their function and molecular role were not yet known.^5,6^ We now know that the *TSC1* and *TSC2* genes encode for hamartin (TSC1) and tuberin (TSC2), which form a regulatory complex responsible for limiting the activity of an important intracellular regulator of cell growth and metabolism known as mammalian target of rapamycin complex 1 (mTORC1) via inhibition of the small GTPase ras homolog enriched in brain (Rheb).^7^ The functional relationship between TSC1/TSC2 and mTORC1 has led to important clinical advances in the use of mTORC1 inhibitors for the treatment of several clinical manifestations of TSC, including cerebral subependymal giant cell astrocytoma,^8–11^ renal angiomyolipomas,^8,12,13^ and pulmonary lymphangioleiomyomatosis (LAM).^8,13–15^ Significant advances in imaging, surgery, interventional radiology, medical, and behavioral therapies have transformed TSC management since 1998.

The extent of medical advances in TSC and the need to standardize and optimize clinical care for individuals with TSC necessitated updating the diagnostic criteria and clinical management guidelines from 1998. In 2011, the International Tuberous Sclerosis Complex Consensus Conference was organized and sponsored by the Tuberous Sclerosis Alliance, a nonprofit patient advocacy group and member of Tuberous Sclerosis Complex International (TSCi). Identification of disease focus areas, participating clinical expert contributors, clinical questions to address, literature review process, and draft recommendations followed. On June 14–15, 2012, 79 experts from 14 countries convened in Washington, DC, to finalize diagnostic, surveillance, and management recommendations for patients with TSC. Finishing work and editing continued into early 2013. A summary report of revised diagnostic criteria for TSC is provided separately.^16^ Here we summarize the updated surveillance and management recommendations for the standardized, optimal clinical management of patients with TSC.

## Methods

Twelve subcommittees, each led by a clinician with advanced expertise in TSC and the relevant medical subspecialty, were organized to focus on specific disease focus topics that have important clinical management implications in TSC: (1) dermatology and dentistry; (2) nephrology; (3) pulmonology; (4) cardiology; (5) ophthalmology; (6) gastroenterology; (7) endocrinology; (8) genetics; (9) epilepsy; (10) TSC-associated neuropsychiatric disorders; (11) brain structure, tubers, and tumors; and (12) coordination of clinical care. Each subcommittee was charged with formulating key clinical questions to address within its focus area, reviewing relevant literature, evaluating the strength of data, and providing a recommendation based on evaluated literature or, if data were lacking, an expert opinion based on experience or case studies or other appropriate method. If no recommendation could be provided because there was no consensus or conflicting evidence was found of equal value or weight, the subcommittee was to provide recommendations for future research that would help resolve the conflict.

A centralized literature search was performed on March 12, 2012, for all consensus group subcommittees to use. This search used PUBMED and SCOPUS databases of all articles published between 1997 (year before last consensus conference) and 2012 (current), regardless of language. Search terms for PUBMED consisted of “tuberous sclerosis” and “humans” and “diagnosis OR therapy.” Search terms for SCOPUS consisted of “tuberous sclerosis” and “diagnosis OR treatment.” A total of 2692 articles were identified with this approach. Each consensus group subcommittee was then able to determine additional terms pertinent to its organ system or disease focus area to further refine articles to be reviewed and evaluated. Additional literature searches, if deemed necessary by individual subcommittees to address key clinical questions not captured by the central literature search, could be performed as needed (e.g., epilepsy surgery or organ transplantation guidelines relevant but not specific to TSC).

The evidence-based framework based on the approach of the National Comprehensive Cancer Network (NCCN) Clinical Guidelines^17^ was used to grade strength of evidence and resulting recommendations. The NCCN framework allows recommendations based on all classes of evidence by categorizing recommendations with regard to the type and strength of evidence used to support the recommendation and is well-suited for application across many organ systems and specialties for a rare disease such as TSC with multisystem involvement. NCCN Clinical Guidelines category 1 recommendations are based on high-level evidence and uniform consensus, whereas category 2 recommendations are based on lower-level evidence and either uniform consensus or consensus. Category 3 recommendations are those for which a consensus cannot be reached, regardless of evidence. Additional details regarding this framework, including definitions for high- and low-level evidence, are provided in Table 1.

For the purposes of this summary document, the 2012 International Tuberous Sclerosis Complex Consensus Group surveillance and management recommendations are organized into two sections: (1) recommendations applicable at the time of initial diagnosis and (2) recommendations applicable to follow-up health care. There is some overlap with this approach because some features discovered upon initial diagnosis may require immediate intervention, additional workup, or specialist referral. By necessity, discussion in this summary is limited to the most relevant and salient points. More detailed discussion of specific recommendations for the different TSC disease focus areas, supporting evidence thereof, and other special considerations will be published separately by each International Tuberous Sclerosis Consensus Complex Group subcommittee.

## Surveillance and management recommendations for individuals with newly suspected or newly diagnosed TSC

TSC is usually first suspected in individuals when one or more clinical diagnostic criteria are identified (Table 2). The purposes of initial diagnostic studies are to confirm the diagnosis in individuals with “possible” TSC and to determine the extent of disease and organ involvement in individuals with “definite” TSC. Baseline studies are also important in guiding treatment decisions should additional disease manifestations emerge in later years.

### Genetics

All individuals should have a three-generation family history obtained to determine if additional family members are at risk of diagnosis. Gene testing is recommended for genetic counseling purposes or when the diagnosis of TSC is suspected or in question but cannot be clinically confirmed (Category 1).

### Brain

All individuals suspected of having TSC, regardless of age, should undergo magnetic resonance imaging (MRI) of the brain with and without gadolinium to assess for the presence of cortical/subcortical tubers, subependymal nodules (SEN), other types of neuronal migration defects, and sub-ependymal giant cell astrocytomas (SEGA). If MRI is not available or cannot be performed, computed tomography (CT) or head ultrasound (US) (in neonates or infants when fontanels are open) may be used, although results are considered suboptimal and will not always be able to detect abnormalities revealed by MRI.^18,19^ (Category 1)

During infancy, focal seizures and infantile spasms (IS) are likely to be encountered,^20,21^ and parents should be educated to recognize these even if none have occurred at time of first diagnosis. All pediatric patients should undergo a baseline electroencephalograph (EEG), even in the absence of recognized or reported clinical seizures. (Category 2A)

If the baseline EEG is abnormal, especially when features of TSC-associated neuropsychiatric disorders (TAND) are also present, this should be followed up with a 24-hour video EEG to assess for electrographic or subtle clinical seizure activity. (Category 3)

TAND is new terminology proposed to describe the interrelated functional and clinical manifestations of brain dysfunction common in TSC, including aggressive behaviors, autism spectrum disorders, intellectual disabilities, psychiatric disorders, and neuropsychological deficits as well school and occupational difficulties.^22^ All patients should receive a comprehensive assessment at diagnosis to determine a baseline for future evaluations and to identify areas requiring immediate or early intervention. Comprehensive assessment is likely to require multidisciplinary involvement and clinical teams should maintain a low threshold to initiate early interventions and other management strategies. (Category 1)

Parents of school-going age should be considered for an individual education plan (IEP) based on the individual TAND profile. (Category 2A)

### Kidney

At the time of diagnosis, abdominal imaging should be obtained regardless of age. As for brain, MRI is the preferred modality for evaluation of angiomyolipomata because many can be fat-poor and hence missed when abdominal CT or US are performed.^23^ MRI of the abdomen may be combined in the same session as MRI of the brain, thereby limiting the need for multiple sessions of anesthesia if anesthesia is needed for successful MRI. MRI of the abdomen may also reveal aortic aneurysms or extrarenal hamartomas of the liver, pancreas, and other abdominal organs that also can occur in individuals with TSC. In addition to imaging, accurate blood pressure assessment is important because of increased risk of secondary hypertension. To assess renal function at time of diagnosis, blood tests to determine glomerular filtration rate (GFR) using creatinine equations for adults^24,25^ or children.^26^ Alternatively, measurement of serum cystatin C concentration can be used to evaluate GFR.^27^ (Category 1)

### Lung

To evaluate for LAM, females 18 years or older should have baseline pulmonary function testing, 6-minute walk test, and high-resolution chest computed tomography (HRCT). When possible, low-radiation protocols should be used. A serum vascular endothelial growth factor type D (VEGF-D) level may be helpful to establish a baseline for future LAM development or progression.^28,29^ Counseling on smoking risks and estrogen use (such as some oral contraceptive preparations), which can compound the impact of LAM, should also occur in adolescents and adults. (Category 2A)

### Skin and teeth

All patients should undergo a detailed clinical dermatologic and dental exam at time of diagnosis to evaluate for facial angiofibromas, fibrous cephalic plaques, hypomelanotic macules or confetti lesions, ungual fibromas, shagreen patch, defects in tooth enamel, and intraoral fibroma. (Category 2A)

### Heart

In pediatric patients, especially younger than three years of age, an echocardiogram and electrocardiogram (ECG) should be obtained to evaluate for rhabdomyomas and arrhythmia, respectively. In those individuals with rhabdomyomas identified via prenatal ultrasound, fetal echocardiogram may be useful to detect those individuals with high risk of heart failure after delivery. (Category 1)

In the absence of cardiac symptoms or concerning medical history, echocardiogram is not necessary in adults, but as conduction defects may still be present and may influence medication choice and dosing,^30^ a baseline ECG is still recommended. (Category 2A)

### Eye

A baseline ophthalmologic evaluation, including funduscopic evaluation, is recommended for all individuals diagnosed with TSC to evaluate for hamartomas and hypopigmented lesions of the retina. (Category 1)

### Other

Although vascular aneurysms, gastrointestinal polyps, bone cysts, and various endocrinopathies can be associated with TSC, there is insufficient evidence to support routine evaluation at time of diagnosis unless there are clinical symptoms or other concerning history that warrants specific investigation. (Category 3)

## Ongoing surveillance and management recommendations for individuals previously diagnosed with TSC

Once the diagnosis of TSC is established and initial diagnostic evaluations completed, continued surveillance is necessary to monitor progression of known problems or lesions and emergence of new ones (Table 3).^20^ Some manifestations begin in childhood and are less likely to be present or cause new problems in adulthood, such as cardiac rhabdomyomas or subependymal giant cell astrocytomas. In contrast, problems with LAM are typically limited to adults, and renal manifestations require significantly more monitoring and intervention in adulthood compared with childhood because of the cumulative nature of angiomyolipomata and other renal lesions. Finally, other aspects of TSC may be present throughout the entire lifespan of the individual, such as epilepsy and TAND, but specific manifestations and impact on overall health and quality of life can vary. Thus, ongoing periodic surveillance is needed after initial diagnosis for optimal care and prevention of secondary complications associated with TSC. Management of specific complications of TSC will often require input from a multidisciplinary team.

### Genetics

Genetic testing and counseling should be offered to individuals with TSC when they reach reproductive age, and first-degree relatives of affected individuals should be offered clinical assessment and, where a mutation has been identified in the index case, genetic testing. (Category 1)

### Brain

Symptomatic SEGA or SEGA associated with increasing ventricular enlargement, or with unexplained changes in neurological status or TAND symptoms, require intervention or more frequent clinical monitoring and reimaging. For acutely symptomatic individuals, surgical resection is the recommended intervention, and cerebrospinal fluid diversion may also be necessary. For growing but otherwise asymptomatic SEGA, either surgical resection or medical therapy with mTOR inhibitors can be effective.^31,32^ Shared decision-making with the patients or their parents in selecting the best treatment option should take the following considerations into account: risk of complications or adverse effects, cost of treatment, expected length of treatment, and potential impact on TSC comorbidities. Patients with unilateral, single, gross total resectable SEGA without individual risk factors or other comorbidities preferentially may benefit from surgery, whereas patients with multisystem disease or multiple or infiltrating SEGA lesions that are not amenable to gross total resection may favor mTOR inhibitor treatment. (Category 1)

Optimal outcome is associated with early detection and treatment,^33^ so surveillance by MRI should be performed every 1–3 years in all individuals with TSC until the age of 25 years. The frequency of scans within the recommended range of every 1–3 years should be clinically determined, with scans performed more frequently in those asymptomatic SEGA patients who are younger, whose SEGA are larger or growing, or who are developmentally or cognitively disabled such that they cannot reliably report subtle symptoms. (Category 2A)

Individuals without SEGA by the age of 25 years do not need continued surveillance imaging, but those with asymptomatic SEGA present in childhood should continue to be monitored by MRI for life because of the possibility of growth. There is insufficient evidence to determine the recommended frequency of MRI surveillance in this latter group, but important clinical factors that would favor shorter intervals include SEGA with proximity to foramen of Monro, large size, or recently discovered. However, once stability is clearly established, it may be possible to increase the interval of surveillance monitoring over time. (Category 3)

Strong evidence demonstrates superior efficacy for the treatment of infantile spasms with vigabatrin in patients with TSC^34–37^; therefore, vigabatrin should be first-line treatment. However, the prescribing clinician should be aware of possible side effects, particularly possible retinal toxicity, and how to monitor for these. Adrenocorticotropin hormone (ACTH) can be used as second-line therapy if treatment with vigabatrin fails. (Category 1)

Routine EEG is recommended in individuals with known or suspected seizure activity, but frequency should be determined by clinical need rather than a specific defined interval. If changes in sleep, behavior, or cognitive or neurological function are not explained by routine EEG, 24-hour video EEG should be considered to assess for unrecognized or subclinical seizure activity. (Category 2A)

Early epilepsy treatment may be of benefit in infants and children during the first 24 months of life if ictal discharges occur, with or without clinical manifestations.^38^ Other than for infantile spasms in TSC, there is little evidence to guide specific anticonvulsant treatment. In general, this should follow that of other epilepsies, but it should be noted that the prevalence of medically refractory epilepsy is high in TSC even with adequate trials of currently available anticonvulsant medications.^30,39^ Epilepsy surgery and vagus nerve stimulation may be considered for medically refractory TSC patients, but evaluation should take place at epilepsy centers with experience and expertise in TSC, and special consideration should be given to children at younger ages experiencing neurological regression. (Category 2A)

Given that the physical features of TSC such as SEGA, epilepsy, or renal failure may present with TAND-like behaviors, sudden and rapid changes in TAND should prompt an urgent overall physical workup in such individuals. (Category 1)

After detailed initial assessment upon diagnosis, it is imperative to continue to monitor for features of TAND and their impact on daily living through basic questioning and screening procedures at each follow-up clinic visit, with a minimum frequency of once per year. Any areas of concern identified at routine TAND assessment should be followed up with more detailed evaluations by the appropriate developmental, neuropsychological, mental health, behavioral, and educational specialists and coordinated by the TSC expert team. (Category 1)

In addition to screening at each clinical visit, comprehensive, formal evaluations for TAND by an expert team should be performed at key scheduled time points: during the first 3 years of life (0–3 year evaluation), preschool (3–6 year evaluation), before middle school entry (6–9 year evaluation), during adolescence (12–16 year evaluation), and in early adulthood (18–25 year evaluation). In later adulthood, evaluations should be performed as clinical challenges emerge or based on TAND screening. More frequent specialty evaluations or treatment/interventions may be needed if annual screening reveals areas of concern. (Category 2A)

Several studies are under way to investigate the use of mTOR inhibitors as treatment for aspects of TAND. To date there is insufficient evidence to support the use of mTOR inhibitors as treatment for any aspects of TAND. There are no other TSC-specific neuropsychiatric interventions to date. However, there is high level evidence of treatment strategies for individual disorders associated with TAND, such as autism spectrum disorder, attention deficit hyperactivity disorder, and anxiety. Clinical teams should therefore use evidence-based principles to guide therapeutic decisions for best treatment of TAND in individuals with TSC, individualized to each patient. (Category 3)

### Kidney

For asymptomatic, growing angiomyolipoma measuring larger than 3 cm in diameter, treatment with an mTOR inhibitor is currently recommended as the most effective first-line therapy in the short term.^8,13,14,40^ The demonstrated tolerability so far to date is far preferable to the renal damage caused by angiomyolipoma progression as well as surgical and embolitic/ablative therapies, though studies are still needed to confirm long-term benefits and safety. (Category 1)

Annual clinical assessment of renal function and hypertension is required. Blood pressure control is also critical, so accurate measurement of blood pressure for patients is crucial, using age-specific criteria for children.^41^ Patients with hypertension should be treated with an inhibitor of the renin-aldosterone-angiotensin system as first line therapy, but avoiding an angiotensin-converting enzyme inhibitor in those treated with an mTOR inhibitor. (Category 1)

Imaging to diagnose polycystic disease, renal cell carcinoma or other tumors,^42,43^ and changes in angiomyolipoma should also be performed. MRI, often obtainable at the same time as brain surveillance imaging, is the preferred imaging modality, but if MRI is not available, CT or US can still provide useful information.^44^ Selective embolization followed by corticosteroids,^45^ kidney-sparing resection, or ablative therapy for exophytic lesions are acceptable second-line therapy for asymptomatic angiomyolipomata. For acute hemorrhage, embolization followed by corticosteroids is more appropriate.^46^ Nephrectomy is to be avoided because of the high incidence of complications and increased risk of future renal insufficiency, end-stage renal failure, and the poor prognosis that results from chronic kidney disease.^12,47^ Fat-poor angiomyolipomata are not uncommon in patients with TSC, but if there is doubt and lesions are growing faster than 0.5 cm per year,^48^ a needle biopsy using a sheath technique or an open biopsy may be considered. (Category 2A)

### Lung

In individuals at risk for LAM, typically females 18 years of age and older, history at each clinical examination should inquire for symptoms of exertional dyspnea and shortness of breath. In patients with no clinical symptoms and no evidence of lung cysts on their baseline HRCT, repeat HRCT imaging should be performed every 5–10 years, using low-radiation imaging protocols when available. Once cysts are detected, pace of TSC-LAM progression should be determined via HRCT testing every 2–3 years accompanied by annual pulmonary function testing and 6-minute walk test. If many cysts or other evidence of advanced TSC-LAM are present, pulmonary function testing and HRCT may be needed as frequently as every 3–6 months to assist with treatment decision-making. (Category 1)

In select LAM patients with moderate-to-severe lung disease or rapid progression, treatment with an mTOR inhibitor may be used to stabilize or improve pulmonary function, quality of life, and functional performance.^8,13–15^ (Category 1)

TSC-LAM patients are candidates for lung transplantation, but it is important to note that antirejection medications may lower seizure threshold and seizure medications may interfere with antirejection medications. TSC comorbidities could also impact transplant suitability. (Category 2A)

### Skin and teeth

A skin survey should be performed annually, with focus on rapidly changing or symptomatic (problematic or functionally impacting) lesions and using pathological evaluation when required for diagnosis. Early intervention is indicated for bleeding, symptomatic, or potentially disfiguring TSC skin lesions. There is insufficient evidence to guide choice of treatment—case reports and case series document successful use of surgical excision, lasers, and topical mTOR inhibitors.^49–53^ (Category 3)

For TSC-associated dental lesions and oral fibromas, periodic oral evaluation should occur every 3–6 months, consistent with surveillance recommendations for all individuals in the general population. Periodic preventive measures as well as oral hygiene education are important in patient management. Bony jaw lesions (asymmetry, asymptomatic swelling, or abnormal tooth eruption), when present, should be evaluated with a panoramic radiograph and treated with surgical excision or curettage if symptomatic or deforming.^54^ Enamel defects (dental pits) can be treated with restorative treatments if the patient is at high cavity risk, although they rarely cause symptoms or an increased incidence of dental decay.^55,56^ Oral fibromas should be excised surgically if symptomatic or if interfering with oral hygiene. Oral fibromas may recur once excised; therefore, periodic oral evaluation is encouraged.^57^ (Category 3)

### Heart

Until regression of cardiac rhabdomyomas is documented, follow-up echocardiogram should be performed every 1–3 years in asymptomatic patients. In addition, 12-lead ECG is recommended at minimum every 3–5 years to monitor for conduction defects. In patients with clinical symptoms, additional risk factors, or significant abnormalities on routine echocardiogram or ECG, more frequent interval assessment may be needed and may include ambulatory event monitoring. (Category 1)

### Eye

Individuals with no identified ophthalmologic lesions or vision symptoms at baseline, reevaluation is necessary only if new clinical concerns arise. Otherwise, annual evaluation is recommended. For patients on vigabatrin, ophthalmologic evaluation every 3 months is recommended by the United States Food and Drug Administration, although utility of such frequent assessment is questioned, especially in the young and those with developmental disability that limit the extent of ophthalmologic evaluation that can be performed.^30,58^ Thus, even in these populations, annual ophthalmologic evaluation is considered more appropriate. (Category 2B)

### Other

There is limited, low-level evidence to guide recommendations for gastrointestinal, endocrine, and other hamartomatous lesions associated with TSC. Follow-up imaging to ensure stability of these lesions, when present, is recommended. Biopsy of suspicious lesions is recommended only when lesions are unusually large, growing, functional, symptomatic, multiple, or exhibit other suspicious characteristics. (Category 3)

## Coordination of care and other clinical considerations in patients with TSC

TSC is a heterogeneous genetic disorder with variable expression and thus its clinical presentations are protean. The primary pathology of concern is also different depending on the age of the affected individual. The involvement of multiple organ systems, at different stages in life, presents major difficulties in locating and identifying the expertise to comprehensively manage the medical care of individuals with TSC. The purpose of the 2012 International TSC Consensus Conference was to provide recommendations that help standardize the approach to managing TSC regardless of age or severity of the disease. Currently in the United States and many other countries, specialized TSC clinics have been established. Ideally, all TSC patients would have access to these clinics to ensure the appropriateness of care and treatment, but this ultimately may not be possible. In circumstances in which individuals with TSC do not have access to the specialized TSC clinics, the recommendations from the TSC Consensus Conference will be of significant value. Another source of invaluable information would be prominent advocacy groups such as the Tuberous Sclerosis Alliance in the United States and many similar groups in countries throughout the world who are also members of Tuberous Sclerosis International.

Resources must be used efficiently, particularly when there are financial or technological limitations. Transition clinics or clinics/facilities that treat both children and adults with TSC are important, particularly for the more severely affected and those with multiorgan system effects. Doing so can avoid duplicative tests and services and ensure appropriate surveillance and symptom management is in place to prevent more costly medical complications. TSC clinics may be institution-based or community-based using a network of clinicians expert in the different aspects of TSC. These clinics must be able to address the psychosocial challenges that face the individual and their family or caregivers as well as the medical needs.

These diagnostic and surveillance recommendations were developed from an ever-increasing understanding of TSC and supported by published, scientific investigation. Continued improvement in clinical knowledge will likely come from planned and ongoing clinical trials investigating a host of potential treatments for TSC, and also from longitudinal databases (e.g., the US TSC Natural History Database, the TOSCA European TSC Registry), which will serve to capture information on the many manifestations and treatments of TSC throughout the human life cycle. As clinical knowledge of the disease improves, the current recommendations will have to be updated periodically.

## Figures and Tables

**TABLE 1:** Recommendation categories and descriptions

Category	Description	Supporting Evidence
1	Based upon high-level evidence, there is uniform consensus that the intervention is appropriate	At least one convincing class I study OR at least two convincing and consistent class II studies OR at least three convincing and consistent class III studies
2A	Based upon lower-level evidence, there is uniform consensus that the intervention is appropriate	At least one convincing class II study OR at least two convincing and consistent class III studies
2B	Based upon lower-level evidence, there is consensus that the intervention is appropriate	At least one convincing class III study OR at least two convincing and consistent class IV studies
3	Based upon any level of evidence, a consensus on appropriate intervention cannot be reached	Class I–IV studies that are conflicting or inadequate to form a consensus

Class Definitions for Supporting Evidence		
Class I: evidence provided by a prospective, randomized, controlled clinical trial with masked outcome assessment, in a representative population. Class II: evidence provided by a prospective matched group cohort study in a representative population with masked outcome assessment. Class III: evidence provided by all other controlled trials (including well-defined natural history controls or patients serving as own controls) in a representative population, where outcome assessment is independent of patient treatment. Class IV: evidence provided by uncontrolled studies, case series, case reports, or expert opinion.		

**TABLE 2:** Surveillance and management recommendations for newly diagnosed or suspected tuberous sclerosis complex (TSC)

Organ System or Specialty Area	Recommendation
Genetics	Obtain three-generation family history to assess for additional family members at risk of TSC Offer genetic testing for family counseling or when TSC diagnosis is in question but cannot be clinically confirmed
Brain	Perform magnetic resonance imaging (MRI) of the brain to assess for the presence of tubers, subependymal nodules (SEN), migrational defects, and subependymal giant cell astrocytoma (SEGA) Evaluate for TSC-associated neuropsychiatric disorder (TAND) During infancy, educate parents to recognize infantile spasms, even if none have occurred at time of first diagnosis Obtain baseline routine electroencephalogram (EEG). If abnormal, especially if features of TAND are also present, follow-up with a 24-hr video EEG to assess for subclinical seizure activity
Kidney	Obtain MRI of the abdomen to assess for the presence of angiomyolipoma and renal cysts Screen for hypertension by obtaining an accurate blood pressure Evaluate renal function by determination of glomerular filtration rate (GFR)
Lung	Perform baseline pulmonary function testing (pulmonary function testing and 6-minute walk test) and high-resolution chest computed tomography (HRCT), even if asymptomatic, in patients at risk of developing lymphangioleiomyomatosis (LAM), typically females 18 years or older. Adult males, if symptomatic, should also undergo testing Provide counsel on smoking risks and estrogen use in adolescent and adult females
Skin	Perform a detailed clinical dermatologic inspection/exam
Teeth	Perform a detailed clinical dental inspection/exam
Heart	Consider fetal echocardiography to detect individuals with high risk of heart failure after delivery when rhabdomyomas are identified via prenatal ultrasound Obtain an echocardiogram in pediatric patients, especially if younger than 3 yr of age Obtain an electrocardiogram (ECG) in all ages to assess for underlying conduction defects
Eye	Perform a complete ophthalmologic evaluation, including dilated funduscopy, to assess for retinal lesions and visual field deficits

**TABLE 3:** Surveillance and management recommendations for patients already diagnosed with definite or possible tuberous sclerosis complex (TSC)

Organ System or Specialty Area	Recommendation
Genetics	Offer genetic testing and family counseling, if not done previously, in individuals of reproductive age or newly considering having children
Brain	Obtain magnetic resonance imaging (MRI) of the brain every 1–3 yr in asymptomatic TSC patients younger than age 25 yr to monitor for new occurrence of subependymal giant cell astrocytoma (SEGA). Patients with large or growing SEGA, or with SEGA causing ventricular enlargement but yet are still asymptomatic, should undergo MRI scans more frequently and the patients and their families should be educated regarding the potential of new symptoms. Patients with asymptomatic SEGA in childhood should continue to be imaged periodically as adults to ensure there is no growth. Surgical resection should be performed for acutely symptomatic SEGA. Cerebral spinal fluid diversion (shunt) may also be necessary. Either surgical resection or medical treatment with mammalian target of rapamycin complex (mTOR) inhibitors may be used for growing but otherwise asymptomatic SEGA. In determining the best treatment option, discussion of the complication risks, adverse effects, cost, length of treatment, and potential impact on TSC-associated comorbidities should be included in the decision-making process. Perform screening for TSC-associated neuropsychiatric disorders (TAND) features at least annually at each clinical visit. Perform comprehensive formal evaluation for TAND at key developmental time points: infancy (0–3 yr), preschool (3–6 yr), pre-middle school (6–9 yr), adolescence (12–16 yr), early adulthood (18–25 yr), and as needed thereafter. Management strategies should be based on the TAND profile of each patient and should be based on evidence-based good practice guidelines/practice parameters for individual disorders (e.g., autism spectrum disorder, attention deficit hyperactivity disorder, anxiety disorder). Always consider the need for an individual educational program (IEP). Sudden change in behavior should prompt medical/clinical evaluation to look at potential medical causes (e.g., SEGA, seizures, renal disease). Obtain routine electroencephalograph (EEG) in individuals with known or suspected seizure activity. The frequency of routine EEG should be determined by clinical need rather than a specific defined interval. Prolonged video EEG, 24 hr or longer, is appropriate when seizure occurrence is unclear or when unexplained sleep, behavioral changes, or other alteration in cognitive or neurological function is present Vigabatrin is the recommended first-line therapy for infantile spasms. Adrenocorticotropin hormone (ACTH) can be used if treatment with vigabatrin is unsuccessful. Anticonvulsant therapy of other seizure types in TSC should generally follow that of other epilepsies. Epilepsy surgery should be considered for medically refractory TSC patients, but special consideration should be given to children at younger ages experiencing neurological regression and is best if performed at epilepsy centers with experience and expertise in TSC.
Kidney	Obtain MRI of the abdomen to assess for the progression of angiomyolipoma and renal cystic disease every 1–3 yr throughout the lifetime of the patient. Assess renal function (including determination of glomerular filtration rate [GFR]) and blood pressure at least annually. Embolization followed by corticosteroids is first-line therapy for angiomyolipoma presenting with acute hemorrhage. Nephrectomy is to be avoided. For asymptomatic, growing angiomyolipoma measuring larger than 3 cm in diameter, treatment with an mTOR inhibitor is the recommended first-line therapy. Selective embolization or kidney-sparing resection are acceptable second-line therapy for asymptomatic angiomyolipoma.
Lung	Perform clinical screening for lymphangioleiomyomatosis (LAM) symptoms, including exertional dyspnea and shortness of breath, at each clinic visit. Counseling regarding smoking risk and estrogen use should be reviewed at each clinic visit for individuals at risk of LAM. Obtain high-resolution computed tomography (HRCT) every 5–10 yr in asymptomatic individuals at risk of LAM if there is no evidence of lung cysts on their baseline HRCT. Individuals with lung cysts detected on HRCT should have annual pulmonary function testing (pulmonary function testing and 6-min walk) and HRCT interval reduced to every 2–3 yr. mTOR inhibitors may be used to treat LAM patients with moderate to severe lung disease or rapid progression. TSC patients with LAM are candidates for lung transplantation but TSC comorbidities may impact transplant suitability.
Skin	Perform a detailed clinical dermatologic inspection/exam annually. Rapidly changing, disfiguring, or symptomatic TSC-associated skin lesions should be treated as appropriate for the lesion and clinical context, using approaches such as surgical excision, laser(s), or possibly topical mTOR inhibitor.
Teeth	Perform a detailed clinical dental inspection/exam at minimum every 6 months and panoramic radiographs by age 7 yr, if not performed previously. Symptomatic or deforming dental lesions, oral fibromas, and bony jaw lesions should be treated with surgical excision or curettage when present.
Heart	Obtain an echocardiogram every 1–3 yr in asymptomatic pediatric patients until regression of cardiac rhabdomyomas is documented. More frequent or advanced diagnostic assessment may be required for symptomatic patients. Obtain electrocardiogram (ECG) every 3–5 yr in asymptomatic patients of all ages to monitor for conduction defects. More frequent or advanced diagnostic assessment such as ambulatory and event monitoring may be required for symptomatic patients.
Eye	Perform annual ophthalmologic evaluation in patients with previously identified ophthalmologic lesions or vision symptoms at the baseline evaluation. More frequent assessment, including those treated with vigabatrin, is of limited benefit and not recommended unless new clinical concerns arise.

## Appendix. Members of the 2012 International TSC Consensus Group

**Table 4:**

Conference co-chairs:	Hope Northrup MD (Houston, Texas)
	Darcy A. Krueger MD PhD (Cincinnati, Ohio)
TS Alliance representatives:	Steven Roberds PhD (Silver Spring, Maryland)
	Katie Smith (Silver Spring, Maryland)
Genetics chair:	Julian Sampson DM FRCP FMedSci (Cardiff, Wales, UK)
Genetics committee:	Bruce Korf MD PhD (Birmingham, Alabama)
	David J. Kwiatkowski MD PhD (Boston, Massachusetts)
	David Mowat MD (Sydney, New South Wales, Australia)
	Mark Nellist PhD (Rotterdam, The Netherlands)
	Hope Northrup MD (Houston, Texas)
	Sue Povey MD (London, England, UK)
TSC-associated neuropsychiatric disorders chair:	Petrus de Vries MBChB, MRCPsych, PhD (Cape Town, South Africa)
TSC-associated neuropsychiatric disorders committee:	Anna Byars PhD (Cincinnati, Ohio)
	David Dunn MD (Indianapolis, Indiana)
	Kevin Ess MD PhD (Nashville, Tennessee)
	Dena Hook (Silver Spring, Maryland)
	Anna Jansen MD PhD (Brussels, Belgium)
	Bryan King MD (Seattle, Washington)
	Mustafa Sahin MD PhD (Boston, Massachusetts)
	Vicky Whittemore PhD (Bethesda, Maryland)
Epilepsy chair:	Elizabeth Thiele MD PhD (Boston, Massachusetts)
Epilepsy committee:	E. Martina Bebin MD MPA (Birmingham, Alabama)
	Harry T. Chugani MD (Detroit, Michigan)
	Peter Crino MD PhD (Philadelphia, Pennsylvania)
	Paolo Curatolo MD (Rome, Italy)
	Greg Holmes MD (Lebanon, New Hampshire)
	Rima Nabbout MD PhD (Paris, France)
	Finbar O’Callaghan MA, MB, MSc, PhD (Bristol, England, UK)
	James Wheless MD (Memphis, Tennessee)
	Joyce Wu, MD (Los Angeles, California)
Dermatology and dentistry chair:	Thomas N. Darling MD PhD (Bethesda, Maryland)
Dermatology and dentistry committee:	Edward W. Cowen MD MHSc (Bethesda, Maryland)
	Elizabeth Gosnell DMD (Columbus, Ohio)
	Adelaide Hebert MD (Houston, Texas)
	Greg Mlynarczyk DDS (Santa Rosa, California)
	Keyomaurs Soltani MD (Chicago, Illinois)
	Joyce Teng MD PhD (Palo Alto, California)
	Mari Wataya-Kaneda MD PhD (Osaka, Japan)
	Patricia M. Witman MD (Columbus, Ohio)
Nephrology co-chair:	Chris Kingswood MSc FRCP (Brighton, England, UK)
Nephrology co-chair:	John Bissler, MD (Cincinnati, Ohio, USA)
Nephrology committee:	Klemens Budde, MD (Berlin, Germany)
	John Hulbert MD (Edina, Minnesota)
	Lisa Guay-Woodford MD (Washington DC)
	Julian Sampson DM FRCP FMedSci (Cardiff, Wales, UK)
	Matthias Sauter MD (Munich, Germany)
	Bernard Zonneberg, MD PhD (Utrecht, The Netherlands)
Brain structure, tubers, and tumors chair:	Sergiusz JóŸwiak MD PhD (Warsaw, Poland)
Brain structure, tubers, and tumors committee:	Ute Bartels MD MSc (Toronto, Ontario, Canada)
	Moncef Berhouma MD (Lyon, France)
	David Neal Franz MD (Cincinnati, Ohio)
	Mary Kay Koenig MD (Houston, Texas)
	Darcy A. Krueger MD PhD (Cincinnati, Ohio)
	E. Steve Roach MD (Columbus, Ohio)
	Jonathan Roth MD (Tel Aviv, Israel)
	Henry Wang MD PhD (Rochester, New York)
	Howard Weiner MD (New York, New York)
Pulmonology chair:	Francis X. McCormack MD (Cincinnati, Ohio)
Pulmonology committee:	Khalid Almoosa MD (Houston, Texas)
	Alan Brody MD (Cincinnati, Ohio)
	Charles Burger MD (Jacksonville, Florida)
	Vincent Cottin MD (Lyon, France)
	Geraldine Finlay MD (Boston, Massachusetts)
	Jennifer Glass MS (Cincinnati, Ohio)
	Elizabeth Petri Henske MD (Boston, Massachusetts)
	Simon Johnson MD (Nottingham, England, UK)
	Robert Kotloff MD (Philadelphia, Pennsylvania)
	David Lynch MD (Denver, Colorado)
	Joel Moss MD PhD (Bethesda, Maryland)
	Karen Smith MLS (Bethesda, Maryland)
	Jay Rhu MD (Rochester, Minnesota)
	Angelo Taveira Da Silva MD PhD (Bethesda, Maryland)
	Lisa R. Young MD (Nashville, Tennessee)
Cardiology chair:	Timothy Knilans MD (Cincinnati, Ohio)
Cardiology committee:	Robert Hinton MD (Cincinnati, Ohio)
	Ashwin Prakash MD (Boston, Massachusetts)
	Robb Romp MD (Birmingham, Alabama)
Ophthalmology chair:	Arun D. Singh MD (Cleveland, Ohio)
Gastroenterology chair:	Ashish DebRoy MD (Houston, Texas)
Endocrinology chair:	Pei-Lung Chen MD PhD (Taipei, Taiwan)
Care integration chair:	Steven Sparagana MD (Dallas, Texas)
Care integration committee:	Michael D. Frost MD (St. Paul, Minnesota)
